# Technique for decellularization and characterization of rat esophageal extracellular matrix for potential application as a 3D scaffold

**DOI:** 10.1080/21655979.2025.2564563

**Published:** 2025-09-24

**Authors:** Vidhi Mathur, Jahnavy Madhukar Joshi, Sreekumar N C, Raviraja N Seetharam, Kirthanashri S Vasanthan

**Affiliations:** a Manipal Centre for Biotherapeutics Research, Manipal Academy of Higher Education, Manipal, India; b Department of Plastic surgery, Kasturba Medical College, Manipal Academy of Higher Education, Manipal, India

**Keywords:** Detergent based decellularization, histological analysis, DNA quantification, in vitro studies, rat model

## Abstract

Conventional treatment for esophageal defects involves surgical removal of the defect area and implant conduit tissues. There exist morbidities and mortalities associated with the treatment including fistula and leakage leading to compromise in quality of life. The aim of this study was to optimize a method for complete decellularization of rat esophagus and to solubilize the decellularized extracellular matrix (dECM) proteins to evaluate in vitro properties for scaffold fabrication. For decellularization, rat esophagi were decellularized using 3-[(3-cholamidopropyl) dimethylammonio]-1-propanesulfonate (CHAPS) and sodium dodecyl sulfate (SDS) buffers for 6 h and overnight, respectively. Post decellularization, the tissue was characterized for DNA, glycosaminoglycans, and elastin quantification; H&E and Masson’s trichrome staining; scanning electron microscopy; and SDS-PAGE to evaluate the quantity and quality of the obtained dECM. DNA quantification and histological analysis revealed complete decellularization, while the retention of sGAGs and elastin showed the presence of extracellular proteins in the tissue. The SEM analysis revealed proper orientation of the extracellular matrix and significant proteins were retained in the dECM, which will enhance the regenerative potential. The decellularized tissues were biocompatible, exhibited no toxicity and were also soluble, which can be adapted for scaffold fabrication.

## GRAPHICAL ABSTRACT

**Figure:**
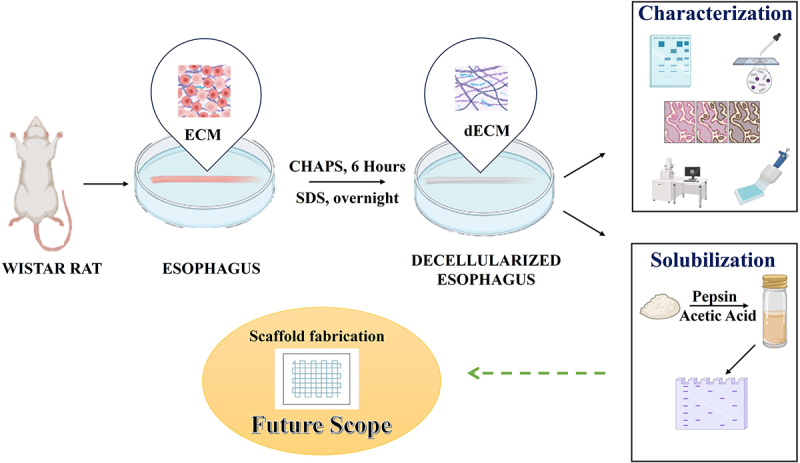

## 1. Introduction

Esophagus is a tubular hollow organ that extends from pharynx to stomach and is made up of four layers, mucosa, submucosa, muscularis, and adventitia and majorly functions for the passage for food [1] There is an increased surge in esophageal disease-related morbidity, affecting more than 10,000 people worldwide [2]. The current gold standard treatment for diseases such as early-stage esophageal malignancies and Barrett’s esophagus includes endoscopic mucosal resection and for addressing severe disorders and end-stage malignancies, esophagectomy procedure is followed [3,4]. Many post-operation complexities are accompanied with the surgery such as anastomosis, strictures, and leakages, due to which there is a high rate of morbidity and mortality [5].

Tissue engineering-based alternatives are the key for the repair and regenerative of esophageal defects, where cultured cells and biomaterials are used in combination to regenerate native tissue *in vitro* [6,7]. Decellularized tissues from animals and humans are routinely used for regeneration, including cardiovascular, skin, bladder, urethra [8]. During the process of decellularization with detergents such as SDS, triton X-100, CHAPS, or enzymes such as trypsin, DNase, and RNase, the cellular contents are completely removed, while they retain the extracellular matrix (ECM) proteins [9]. The ECM is a complex network of proteins and polysaccharides that helps in mimicking the *in vivo* biochemical cues for regeneration and providing structural and biochemical support to cells within tissues [10]. The technique of decellularization significantly reduces the immunogenicity of the decellularized extracellular matrix (dECM) and thus, promotes biocompatibility when compared to scaffolds fabricated only with other biomaterials [11]. Rodent models particularly rats are commonly used for decellularization techniques as they have small size, easy to handle and in physiological relevance [12].

In this study, we have proposed an effective technique of decellularization for harvesting rat esophagus dECM via two-step process using detergents CHAPS and SDS. CHAPS is a zwitterionic detergent and has the properties of both ionic and nonionic detergents and targets lipid–protein interactions and lipid–lipid interactions [13], while SDS, an ionic detergent, helps in the removal of complete cellular contents [14]. Post decellularization, biochemical characterizations such as sGAGs and elastin were quantified along with scanning electron microscopy and histological staining. The solubilization of the decellularized tissue was done with the use of acetic acid and pepsin and post solubilization, the protein content was quantified followed by SDS-PAGE. The solubilized dECM can be used in future as a component of bioink for 3D bioprinting, enhancing the bioactive cues for cells in the 3D bioprinted scaffold [15].

To the best of our knowledge decellularized ECM is used as a scaffold as a recellularization purpose that can be implanted in the host organ but very few work as they lack clarity wherein the dECM is dissolved and completely used for scaffold fabrication. Unlike previous approaches, our method of decellularization focuses on refining the balance between effective cell removal and ECM preservation. By introducing and evaluating an improved technique, the study contributes in generating more biologically relevant components that can be utilized for creating scaffolds [16]. The major challenge lies in dissolving the dECM to fabricate it as a scaffold which is also biocompatible. In this study, we aim to dissolve the tough dECM into a liquid which can be made into different shapes of scaffolds that will be made as an implant for meeting any complications of esophagus disorders. The aim of this paper is to find a suitable agent to dissolve the dECM and make it non-toxic for cells. If established, the same principles can be applied for human esophageal tissues, and the human dECM can be fabricated into various shapes owing to their availability.

## 2. Materials and methods

### 2.1. Materials

CHAPS (CAT # 21420), SDS (CAT#85369), phenol, chloroform (CAT #84155), isoamyl alcohol (CAT #69931), isopropanol (CAT # 62986), and papain (CAT # 95907) were purchased from Sisco Research Laboratories, India. Ethylenediamine tetra acetic acid (EDTA) (CAT#Q37395) and formaldehyde (CAT#Q24005) were procured from Qualigens, India. Glutaraldehyde (CAT#340855) was purchased from Sigma- Aldrich, India. Proteinase K (CAT# 25530049) was received from Invitrogen, India. Coomassie brilliant blue (CAT# 115444)and protease inhibitor cocktail (CAT#539196) was obtained from Merck, India. Sodium acetate (CAT# S0559) was procured from Tokyo Chemical Industry, India. Pierce™ BCA Protein Assay # 23225), Antibiotic-antimycotic (100X) (CAT# 15240062) was purchased from Thermo Fisher Scientific, India, and Blyscan™ - sulfated Glycosaminoglycan (sGAG) assay kit (CAT# 502119981) and Fastin™ Elastin Assay kit (CAT# 502119994) from biocolor, United Kingdom. Dulbecco’s Modified Eagle Medium (DMEM) low glucose (CAT# AL006), fetal bovine serum (FBS) (CAT# RM10679), RIPA buffer (CAT#TCL131), 3-(4,5-dimethylthiazol-2-yl)-2,5-diphenyltetrazolium bromide (MTT) (CAT#TC191), DNA ladder (MBT051) was received from Himedia, India. Oil red O stain (CATt# 102419), alizarin red stain (CAT# TMS008), alcian blue stain blue (CAT# B8438), Insulin transferrin selenium (CAT# I3146), Insulin, 3-Isobutyl-1-methylxanthine (IBMX) (CAT# I5879), l-ascorbic acid (CAT# A4403), glycerol-2-phosphate (CAT#G9422), Indomethacin (CAT# I7378), Collagenase II (CAT# C2-BIOC), glutamine (CAT# 35050061), penicillin/streptomycin solution (CAT## 15240062) were procured from Sigma-Aldrich, India. Transforming growth factor-1β (CAT# PKSH033947), PE Anti-human CD105 flow antibody (CAT# E-AB-F1310D), PE Anti-human CD73 flow antibody (CAT# E-AB-F1242D), PE Anti-human CD34 flow antibody (CAT# E-AB-F1143D), FITC Anti-human CD45 flow antibody (CAT## E-AB-F1137C) were procured from Elabscience Biotechnology Inc, India, and protein ladder (1610374) was procured from Biorad.

### 2.2. Animal ethics approval

All the animals used in this study were approved by the Institutional Animal Ethics Committee (IAEC) and the Manipal Academy of Higher Education, Manipal, which is in accordance with CPSCEA guidelines (IAEC/KMC/72/2022). All the experiments involving the animals were performed following ARRIVE guidelines.

### 2.3. Human ethics approval

The adipose tissue was used for isolation of adipose tissue-derived mesenchymal stem cells, and approval from the Institutional Ethics Committee (IEC) of Kasturba Medical College (KMC) was obtained for this study (IEC/842/2021). All the studies involving human samples adhered to the declaration of Helsinki. The tissue (*n* = 1) was collected from patients undergoing liposuction in the abdomen from the Department of Plastic Surgery, KMC, Manipal. Patients were informed about the study, and consent was taken. Healthy adults under the age of 40 were included in the study and came under inclusion criteria, while patients with diabetes, hypertension, and pregnant women were excluded for the study.

## 3. Methods

### 3.1. Preparation of rat esophagi

Animals were generated by inhouse breeding, as the facility is having license for inhouse breeding (Central Animal Research Facility 94/PO/RReBi/S/99/CPSCEA, dated 29 April 2022) and housed at the Central Animal Facility. Esophagi were harvested under aseptic conditions from male Wistar rats (*Rattus norvegicus*) (9–12 weeks old), weighing 250–300 gby anesthetizing with sodium pentobarbital (50 mg/kg IP). Papaverine (20 mg/kg) was injected into the tail vein to relax the visceral muscles and euthanize the animal as per CPCSEA guidelines. The cervical segment of the esophagus was dissected free from its adjacent tissue. Next, the thoracic and abdominal cavities were opened. After pouring freshly prepared 1X cold Krebs solution into the thoracic cavity, the esophagus was removed and dissected free from adjacent tissues. The entire esophagus was cut at the proximal and distal ends, including the very first part of the stomach and immediately placed in phosphate-buffered saline (PBS) with 1% antibiotic–antimycotic solution. Each harvested esophagus was rinsed in 1× PBS and processed immediately for decellularization. Total of 10 esophagi were harvested from 10 rats for all the experiments.

### 3.2. Decellularization of rat esophagi

For the decellularization process, the rat esophagus was thoroughly cleaned to remove residual tissue and remnants of blood with 1× PBS (pH 7.4). Further, the esophagus was cut into four segments (~1 cm) and suspended in CHAPS buffer (8 mM CHAPS, 1 M NaCl, and 25 mM EDTA in 1× PBS, pH 7) for 6 h at stirring condition (340 rpm) in 2% w/v ratio. Following the treatment, the tissue was washed with 1× PBS and further treated with SDS buffer (1.8 mM SDS, 1 M NaCl, and 25 mM EDTA in 1× PBS, pH 7) and kept overnight at room temperature (24°C) and then further washed once with 1× PBS. This cycle was repeated three times in order to obtain complete decellularized esophagus.

### 3.3. Histological analysis

For histological testing, native and decellularized esophagus samples were fixed for 24 h using 10% formaldehyde and then rehydrated, paraffin embedded, and sectioned with a LEICA RM 2125RT microtome to obtain 3 μm sections. To confirm the absence of genetic material and the presence of ECM proteins, the decellularized samples were stained with Hematoxylin and Eosin (H&E), Masson’s trichrome stain and Alcian blue stain to evaluate the presence of cellular contents, collagen, and glycosaminoglycans, respectively. The stained slides were visualized by using EVOS M5000 microscope, and images were acquired.

### 3.4. Scanning electron microscopy (SEM)

Native esophagi and decellularized tissue samples were fixed in 2.5% glutaraldehyde for 2 h at 4℃and the samples were dehydrated in gradient ethanol from 10% to 100%, with 10 min each in 10–80% and 20 min in 90–100% and then the samples were incubated at 37°C to air dry overnight. The samples were mounted on stubs using carbon coated tape and coated with gold using an ion beam coater (Quorum SC7620 sputter coater 120 s, 10 Pa), and the samples were imaged at 10 kV voltage using Zeiss EVO MA18 SEM, Germany.

### 3.5. Quantification of DNA content

The isolation of genomic DNA was performed using an optimized inhouse protocol with phenol: chloroform: isoamyl alcohol extraction method. About 50 mg of native and decellularized samples was weighed and then homogenized with 1× PBS (pH 7.4) using Scilogex SC16 PRO homogenizer, USA. The samples were centrifuged at 8000 rpm for 10 min and the supernatant was discarded. For the tissue pelleting, lysis buffer, Tris-EDTA buffer, and proteinase K were added with volumes 200 µL, 400 µL and 10 µL, respectively. The samples were vortexed and kept at 55°C until the tissue was completely digested. Post digestion, about 600 µL phenol was added and vortexed for 10 min at 25 rpm, followed by centrifugation at 8000 rpm for 10 min at 4°C. The aqueous layer was pipetted out into a fresh 1.5 mL microcentrifuge tube, and 300 µL phenol and chloroform: isoamyl alcohol (CIA) (24:1; 300 µL) was added, and the samples were vortexed for 10 min at 25 rpm, followed by centrifugation at 8000rpm, for 10 min at 4°C. The aqueous layer was separated via pipetting, and an equal volume of CIA was added, and the samples were vortexed for 30 min at 25 rpm, followed by centrifugation at 8000 rpm, for 10 min at 4°C. The aqueous layer is pipetted out carefully into a fresh microcentrifuge tube that contains 600 µL of chilled isopropanol and 60 µL of sodium acetate and the tube is inverted. The samples were kept in −20°C for half an hour and further centrifuged at 13,000 rpm for 20 min at 4°C. Post centrifugation, the supernatant was removed, and 70% ethanol was added to the pellet and centrifuged at 13,000 rpm for 20 min at 4°C. Ethanol was completely removed from the tube, and the samples were left to air dry overnight at room temperature. The pellet was dissolved in the Tris EDTA buffer, and DNA was quantified by Synergy H1 multi-mode microplate reader, USA, at 260 nm and 280 nm. Absorbance values were normalized to the wet weight of samples, further 1% Agarose gel was prepared for running gel electrophoresis to visualize the presence of DNA in the samples.

### 3.6. Total protein distribution

The extraction of proteins from native and decellularized esophagus tissues was carried out using 1X RIPA and protease inhibitor cocktail to digest the tissue for 30 min. For the quantification of proteins, bicinchoninic acid (BCA) assay was employed following the manufacturer’s protocol, and absorbance was read at 562 nm. Protein concentrations in the samples were calculated in accordance with the standard protein values.

SDS-PAGE resolving and stacking gels are prepared with concentration 8% and 6%, respectively. For the sample preparation, 20 μg/mL of protein was mixed with a loading buffer and reducing agent (NuPAGE, Thermo Fisher Scientific) and heated for 5 min at 95°C. The stacking gel was run at 80 V, and the resolving gel was run at 100 V until the samples completely ran out of the gel. The gel was stained with Coomassie blue and was visualized using Invitrogen iBright cl1500 imaging system, USA.

### 3.7. Quantification of sGAGs and elastin content

The sGAGs quantification was performed in native and decellularized samples using Pierce™ - sulfated Glycosaminoglycan (sGAG) assay kit as per manufacture’s protocol and photometric measurements were recorded at 656 nm using Microplate Reader (Biotek, SH1M) and compared with the standards that provided in the kit.

Fastin Elastin Assay kit was used according to the instructions provided for quantification of elastin, and photometric measurements were quantified at 513 nm using Microplate Reader (Biotek, SH1M). The standard graph was plotted using the elastin, provided in the kit.

### 3.8. Cytotoxicity assay

#### 3.8.1. AT-MSC cell culture and characterization

The adipose tissue was obtained following liposuction surgery at Kasturba Hospital, Manipal, Karnataka. The tissue was transported to the laboratory and washed with saline and 2% antibiotic/antimycotic solution and centrifuged at 900 rpm for 3 min at 37°C. The adipose tissue from the middle fraction was collected in a different tube. 8 mL collagenase II (1 mg/mL) was added to 20 mL of adipose tissue and kept in rotation for 30 min at room temperature and incubated for 15 min at 37°C. Post incubation, the tube was centrifuged at 1200 rpm for 10 min and the lower stromal vascular fraction was collected, and equal volume of complete media (Dulbecco’s modified Eagle medium (DMEM) low glucose, 10% fetal bovine serum (FBS), 1% glutamine, and 1% penicillin/streptomycin solution was added and centrifuged at 1200 rpm for 10 min at 37°C. The pellet was resuspended in 10 mL complete medium and transferred to *T*-25 flask and incubated at 37°C, 5% CO_2_ incubator with 95% humidity. A working cell stock was prepared at passage 4. Passage 6 adipose tissues derived mesenchymal stem cells (AT-MSCs) were seeded in 24-well plate at density of 10,000 cells/well and let adhere for 24 h.

For characterization, trilineage differentiation and surface marker analysis of AT-MSCs was performed according to ISCT criteria [17]. For trilineage differentiation *in vitro*, AT-MSCs were seeded at a seeding density of 30000cells/well in 48-well plate in complete media. After 24 h, the media was changed to respective differentiation media. For adipogenic differentiation, the media contained FBS (10%), insulin (10 µg/mL) IBMX (500 µM), indomethacin (200 µM), dexamethasone (1 µM) in DMEM-high glucose. Osteogenic differentiation, constituted media containing FBS (10%), glycerol-2-phosphate (10 mM), ascorbic acid (50 µg/mL), dexamethasone (100 nM), glutamine supplement in DMEM-high glucose. For the chondrogenic differentiation, DMEM composition has FBS (10%), insulin transferrin selenium, ascorbic acid (50 µg/mL), dexamethasone (100 nM), transforming growth factor-β1 (10 ng/mL) into DMEM-high glucose. Differentiation was confirmed after 14 days for adipogenesis and osteogenesis, and 21 days for chondrogenesis by staining the extracellular matrix of the differentiated cells. Oil red O staining was performed to confirm adipogenic differentiation, alizarin red staining was performed to confirm osteogenic differentiation, and Alcian blue staining was performed to confirm chondrogenic differentiation.

To perform immunophenotyping, the surface markers of isolated AT-MSCs were analyzed using flow cytometry to assess the levels of CD45, CD34, CD105, and CD73. Flow cytometer antibodies PE conjugated anti-CD34, anti-CD105, anti-CD73, FITC-conjugated anti-CD45 were used. Isolated cells were washed with 1× PBS, incubated with 5 µL fluorophore-stained primary antibody (0.2 mg/mL) for 30 min, and washed using 1× PBS to remove excess unbound antibodies, and were acquired and analyzed using BD Accuri C6 Plus flow cytometer with standard configuration of 533/30 filter in FL1, 585/40 in FL2, 670 long pass in FL3, and 675/25 in FL4.

#### 3.8.2. Preparation of conditioned culture media for assessing cytotoxicity of decellularized tissues

The decellularized tissue was sterilized with 2% antibiotic/antimycotic under agitation at room temperature (24–26℃) for 72 h followed by washing in PBS for 72 h under agitation. Post sterilization, decellularized tissue was incubated with AT-MSC cell proliferation media (100 mg tissue per milliliter) for 72 h at 37°C, 5% CO_2_ incubator with 95% humidity. The conditioned media was then used to culture AT-MSC, and their cell viability was assessed by MTT assay.

#### 3.8.3. Cell viability assay

To analyze the cell viability, three groups were considered: decellularized tissue conditioned media and AT-MSC cultured in DMSO:DMEM (1:1) was the positive control, while AT-MSC cultured in DMEM were negative control (untreated cells). The cell viability of decellularized esophageal tissues was assessed for 72 h using conditioned media for the culture of AT-MSC. After incubating for 72 h, without any media change, MTT assay was performed to determine the cell viability. In all the groups, the media was removed and 500 μL of 0.5 mg/mL MTT was added and incubated for 2.5 h and then the formazan crystals were dissolved in 500 μL DMSO for 15 min in shaking conditions. The optical density was measured at 570 nm by a multimode plate reader. Results were expressed as a percentage of cell viability in treated samples versus untreated cells (100% viability).

### 3.9. Solubilization of decellularized tissue

The decellularized tissue is lyophilized and then further crushed into smaller pieces and is treated with 0.5 M acetic acid containing pepsin (10%w/w) for 72 h at 350 rpm. Pre-solubilization, the pH was 1–1.5, and post-solubilization the pH was neutralized to 7.2–7.5 by adding 10 M NaOH solution.

To assess the protein retention post-solubilization, 1%–2% of solubilized dECM was used for SDS-PAGE and BCA assay. For the quantification of proteins, ELISA-based BCA assay following the manufacturer’s protocol and absorbance was observed at 562 nm. Protein concentrations in the samples were calculated in accordance with the standard protein values.

SDS-PAGE resolving and stacking gels are prepared with concentrations of 10% and 4%, respectively. For the sample preparation, 20 μg/mL of protein was mixed with a loading buffer and reduction agent (NuPAGE, Thermo Fisher Scientific). Protein samples are heated for 5 min at 95°C. The stacking gel was run at 80 V, and the resolving gel was run at 100 V until the samples were completely run out of the gel. The gel was stained with Coomassie blue and was visualized using Invitrogen iBright cl1500 imaging system, USA.

### 3.10. Statistical analysis

All statistical analyses were performed with the aid of GraphPad Prism version 9.5.1 [18]. All the quantitative data were reported as mean ± standard deviation (SD). T-test was applied for all the characterization tests. Significance was set as *p* < 0.05.

## 4. Results

### 4.1. Decellularization of rat esophagus

**Figure 1.**
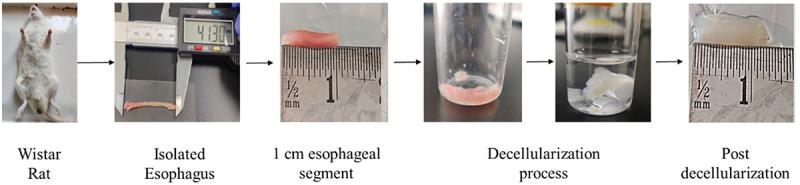
Decellularized rat esophagus post treatment.

The dissected rat esophagus obtained from the male Wistar rat was cleaned with PBS and cut into segments of 1 cm for the decellularization process. The esophagus was decellularized via chemical treatment using CHAPS and SDS for complete removal of cellular content. The macroscopic observation demonstrated the change in tissue color from red (native) to translucent (decellularized) as shown in Figure 1. The appearance of translucent tissue after the process of decellularization indicates the removal of various population of cells from the rat esophagus.

**Figure 2.**
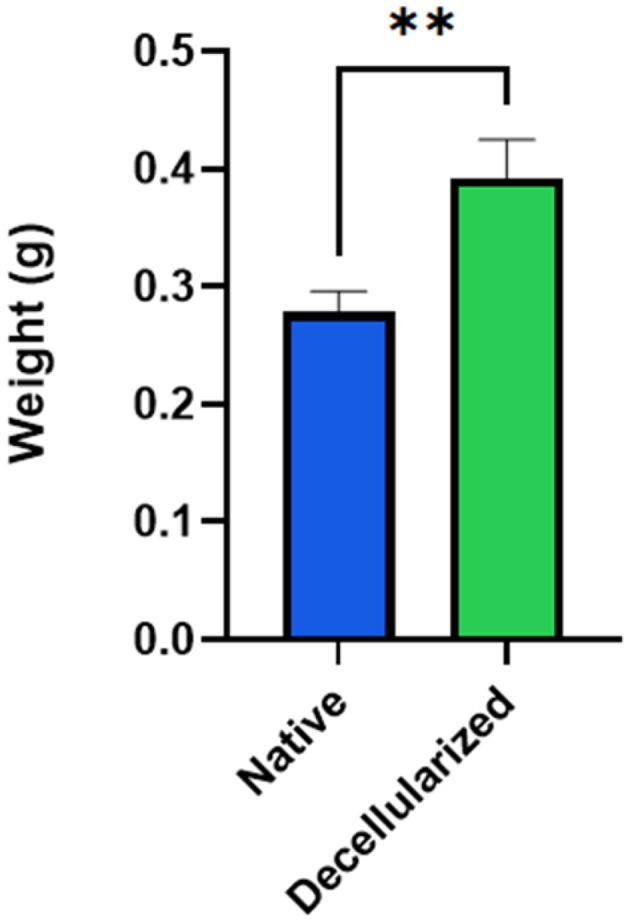
The weight change observed in native and decellularized tissue of rat esophagus. (*p*<0.01).

The weight of rat esophagus was noted before and after decellularization, and it was observed that there was an increase in wet weight in decellularized tissue. The weight of native and decellularized tissue was noted as 0.278 ± 0.014 g and 0.391 ± 0.027 g, respectively (Figure 2).

### 4.2. Scanning electron microscopy

**Figure 3.**
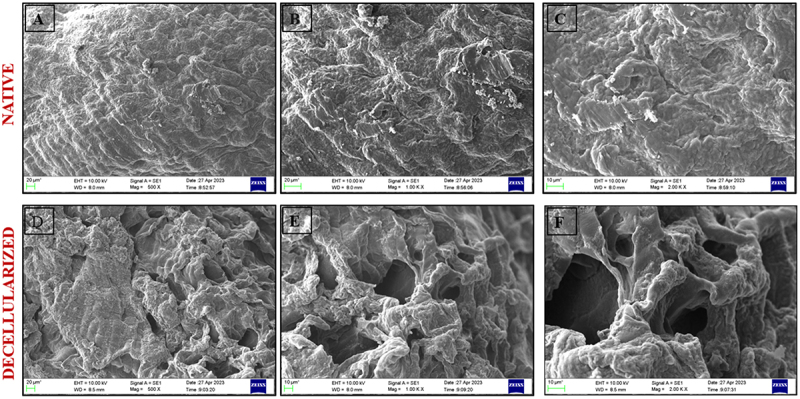
Scanning electron micrographs (A–C) native rat esophagus at magnification 500×, 1000×, and 2000× (D,E) decellularized rat esophagus at magnification 500×, 1000×, and 2000×.

To determine the architectural changes in the tissue post decellularization, SEM images were recorded in both native and decellularized. It was observed that the micro architecture of decellularized tissue contains numerous pores as compared to native tissue, as presented in Figure 3.

### 4.3. Histological analysis

**Figure 4.**
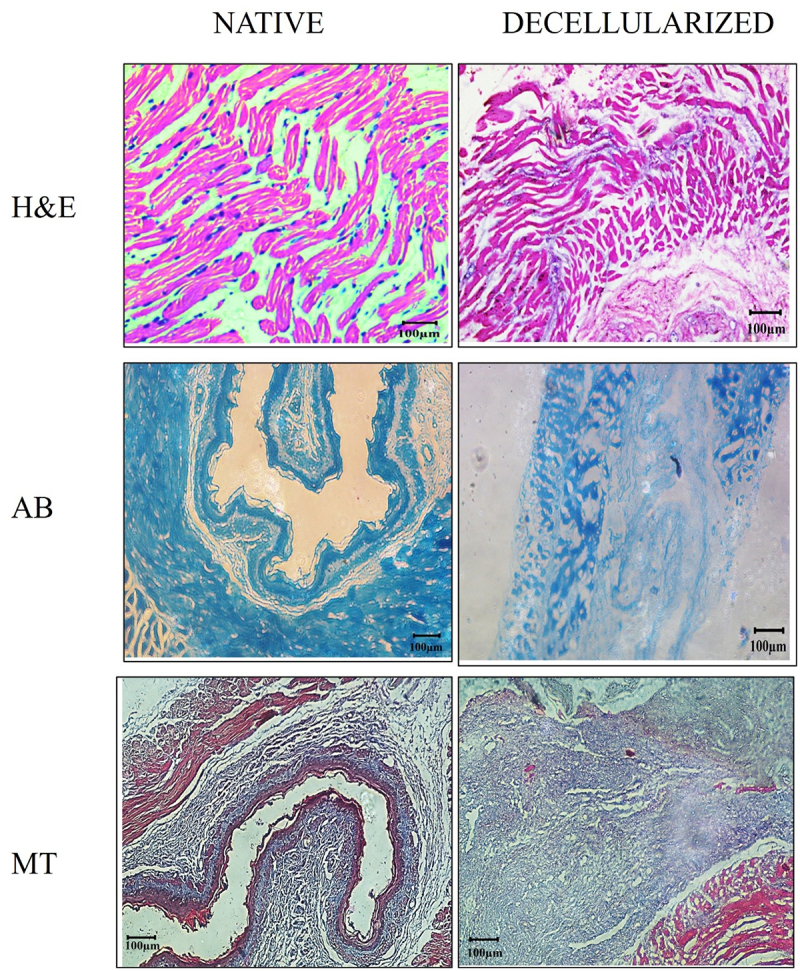
Histological analysis of native and decellularized esophagus using various stain.

The removal of cellular content and preservation of native ECM structure was assessed by Hematoxylin and Eosin (H&E) staining, which showed the absence of violet stained (hematoxylin) nuclear content in the decellularized esophagus. The appearance of the pink color suggests an eosin-stained extracellular matrix. Alcian Blue (AB) and Masson’s trichrome (MT) staining revealed the presence and retention of glycosaminoglycans and collagen, respectively, the prominent ECM proteins of the esophagus (Figure 4).

### 4.4. DNA quantification

**Figure 5.**
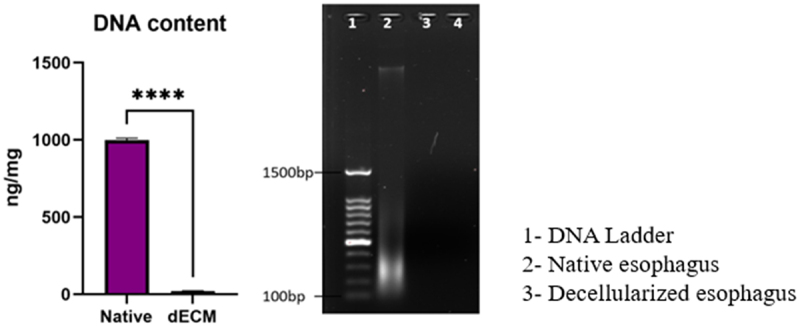
DNA quantification and agarose gel electrophoresis (****p<0.0001).

The absence of cellular content in the decellularized tissue was confirmed by DNA quantification as shown in Figure 5. The residual content of DNA in native and decellularized esophagus was 999.225 ± 10.196 ng/mg and 20.66 ± 2.494ng/mg, respectively. The DNA content of the decellularized tissue was reduced 97.93% from that of native tissue, the values are expressed as mean ± SD (n = 5). Agarose gel electrophoresis results show the absence of bands in the decellularized tissue lane, while a strong dense band in the native tissue lane, confirming the elimination of DNA in the decellularized tissue.

### 4.5. Total protein distribution

**Figure 6.**
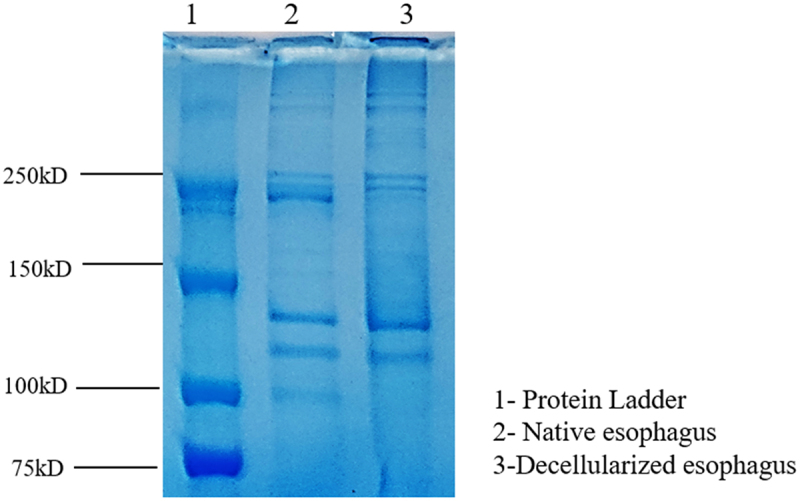
SDS-PAGE to determine the protein distribution in native and decellularized esophagus tissue.

SDS-PAGE was performed to determine the protein distribution and retention by the decellularized esophagus tissue. Figure 6 showcases the presence of protein bands in native and decellularized tissue at varying sizes.

### 4.6. Sulphated glycosaminoglycans (sGAGs) and elastin quantification

**Figure 7.**
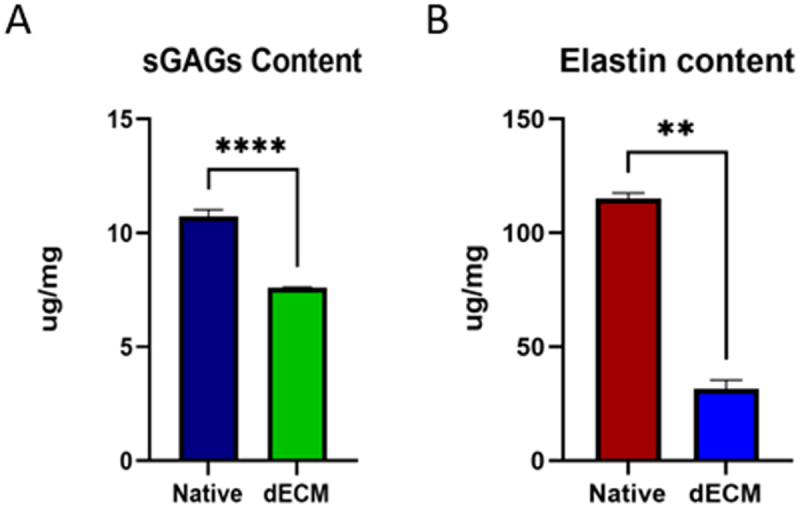
Quantification of A- sGAGs; B- Elastin in native and decellularized esophagus (**p<0.01, ****p<0.0001).

The sGAGs content was retained significantly in native and decellularized tissue. The sGAGs content in native 10.73 ± 0.232 µg/mg of wet tissue and decellularized tissue had 7.587 ± 0.022 µg/mg of wet tissue. The values are expressed as mean ± SD, with a sample size of 05 in each group (Figure 7(A)).

The elastin content in decellularized esophagus tissue was significantly reduced in comparison to native esophagus tissue. The elastin content in native and decellularized tissue were 115.034 ± 1.804 µg/mg of wet tissue and 31.59 ± 2.73 µg/mg of wet tissue, respectively. The values are expressed as mean ± SD, with a sample size of 05 in each group (Figure 7(B)).

### 4.7. AT-MSC characterization

**Figure 8.**
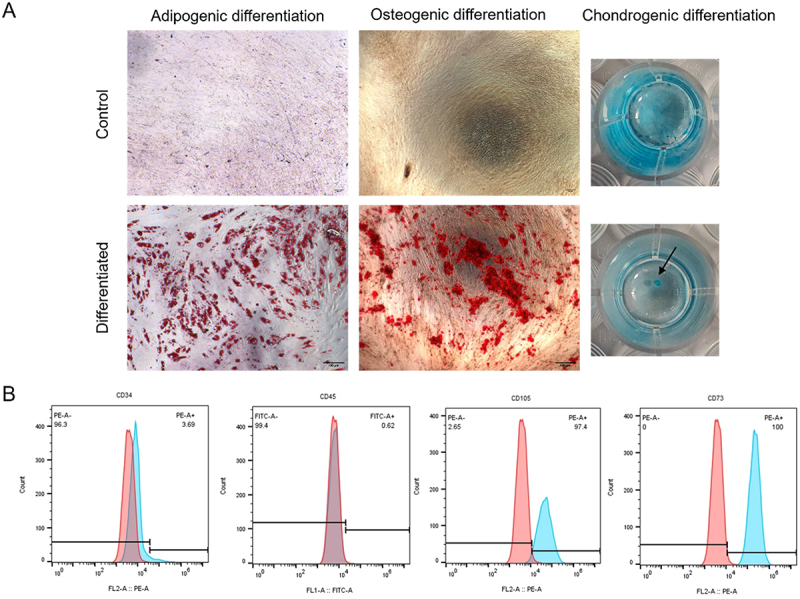
Characterization of AT-MSCs. (A) Staining images of trilineage differentiation by oil red o (adipogenic), alizarin red (osteogenic) and Alcian blue (chondrogenic) of control and differentiated cells; (B) immunophenotyping of AT-MSCs at passage 6.

Isolated AT-MSCs were characterized according to ISCT Criteria by performing trilineage differentiation and immunophenotyping. The AT-MSCs underwent adipogenic, osteogenic and chondrogenic differentiation pertaining to ISCT guidelines. CD34 and CD45 showed less than 5% expression, which are the negative markers. CD105 and CD73 are positive markers which showed more than 95% expression, which fulfilled the ISCT criteria (Figure 8).

### 4.8. Cytotoxicity assay

**Figure 9.**
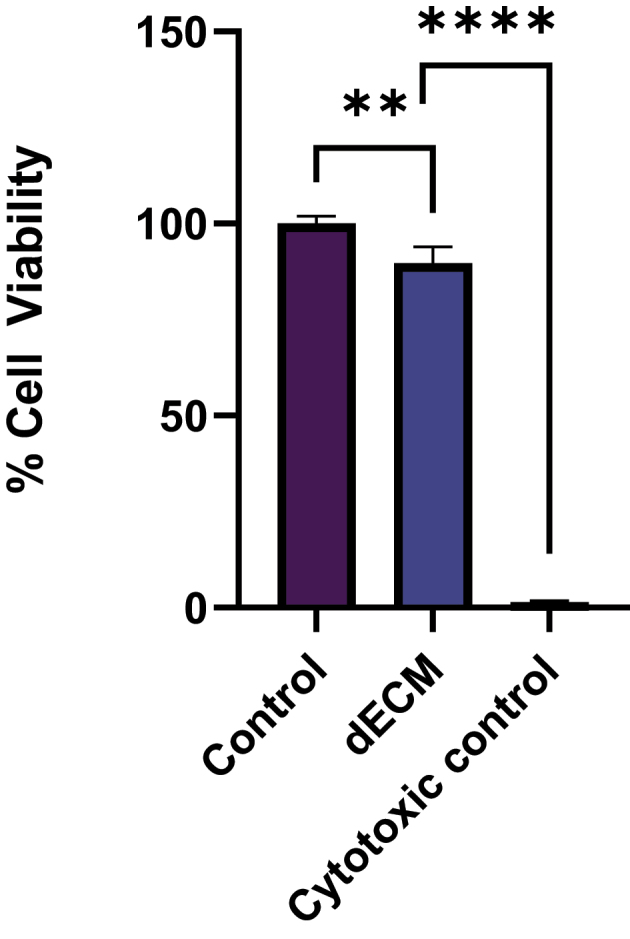
Cytocompatibility study (**p<0.01, ****p<0.0001).

After 72 h of exposure period to conditioned media, AT-MSC appeared to be viable and proliferating, reaching about 90% of confluency. The MTT assay revealed that, cells preserved 89% viability when treated with a medium conditioned with decellularized esophageal matrices when compared to untreated cultures. Significant difference (p < 0.0001) was observed between cytotoxic control and dECM groups, suggesting that cell cultures respond appropriately in comparison with the positive control (Figure 9).

### 4.9. Solubilization and protein retention

**Figure 10.**
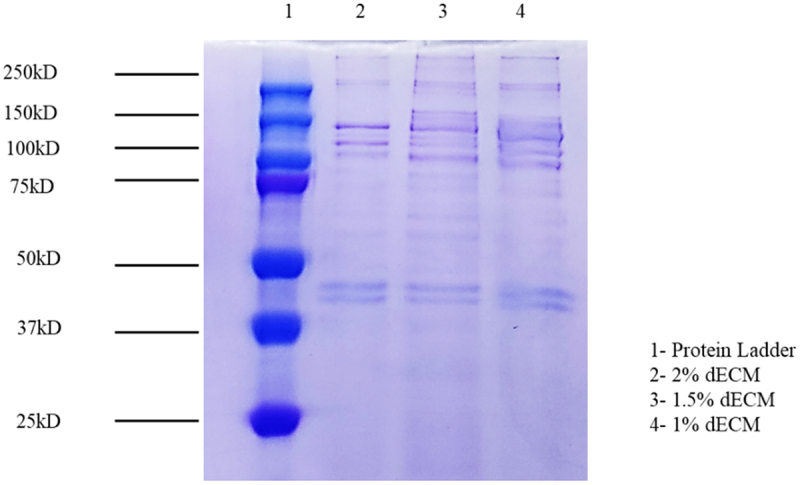
SDS-PAGE to determine the protein retention in solubilised dECM.

SDS-PAGE was performed to determine the protein distribution and retention by the solubilized dECM. Figure 10 showcases the presence of protein bands in 1%, 1.5%, 2% solubilized dECM at varying sizes.

## 5. Discussion

The human esophagus lacks the ability to regenerate and various conventional methods used to treat the esophageal defect includes gastric pull up, primary closure, colon interposition which leads to complications and involves high failure risk and fatal morbidities and mortalities [19]. Artificial grafts for esophagus replacement with similar tubular architecture are potential alternatives, where the immunogenicity and biocompatibility are to be addressed [20]. Artificial grafts made from synthetic polymers or composite materials may not fully replicate the biochemical and biomechanical properties of the native esophagus. They lack an array of bioactive molecules such as ECM proteins and growth factors that are necessary for effective cellular interactions, adhesion, and proliferation. They can exhibit poor biocompatibility that can lead to inflammation and immune responses. In contrast, natural scaffolds that are derived from decellularized tissues provide native microenvironment with the required bioactive cues and ECM proteins [21]. The requirement of esophageal dECM is the need of hours for fabricating scaffolds for repair and regeneration of esophageal defects. Decellularization facilitates the removal of cellular content whilst retaining the ECM proteins such as collagen, GAGs, elastin, and fibronectin. These proteins contribute to tissue repair, regeneration, and cell remodeling and also provide a native microenvironment for cell proliferation. The process of decellularization also eliminated the HLA antigens in the dECM due to the use of detergents, and hence the decellularized tissue turned biocompatible and non-immunogenic [22]. There are certain gold standard criteria to prove the efficacy of the dECM, which includes the DNA quantity in the dECM should measure less than 50 ng/mg of the tissue, the DNA bands observed (if any) in agarose gel should be less than 200 bp [23], and no cellular contents to be observed in Hematoxylin & Eosin/DAPI staining [24].

In this study, we aim to provide an efficient method for decellularizing the rat esophagus to obtain cell-free liquid dECM containing the protein. Decellularization can be achieved by chemical, enzymatic, and physical methods, and herein we have optimized the use of CHAPS, a zwitterionic detergent, which helps in targeting lipid interactions of tissue and solubilizes the membranes, and SDS, denatures the proteins and helps in solubilizing the membranes, as potent agents for the removal of cells from the esophagus. The role of detergents should also protect ECM proteins from damage and remove genetic material, thus making the dECM compatible.

In the present study, we have obtained translucent tissue post decellularization (Figure 1), which suggests the removal of cells, that are confirmed by various assays. The hydrophilic nature of the ECM proteins in dECM leads to an increase in the tissue weight in the decellularized tissue when compared with native or non- decellularized tissue (Figure 2). This attributes to the swelling property of dECM, which resulted in the weight gain in dECM tissues [25]. The removal of cells leads to void space in decellularized tissue and the pores pave the way for the ease of exchange of PBS adding to the swelling property and enhancing the increase in weight of decellularized tissue.

As observed under SEM, the decellularized tissue showed significant presence of pores with rough architecture at various locations when compared to the native tissue due to the action of detergents that helps in cellular proliferation (Figure 3). The histological stains are performed on native and decellularized tissue to validate the presence of cells and the retention of ECM components. The presence of cells in native samples is well depicted by the hematoxylin stain (violet) in Figure 4(A), while the significant absence of hematoxylin stain on dECM as depicted in Figure 4(B) confirms the success of the decellularization technique. The presence of pink stained eosin shows the distribution of ECM proteins further Figure 4(D) confirms the presence of sGAGs proteins that are stained blue. The Alcian blue stain in native tissue (Figure 4(C)) also helps to compare the distribution of GAG in native and decellularized tissues.

The presence of collagen is demonstrated by Masson’s trichrome stain and appearance of blue color in Figure 4(F) confirms collagen distribution in decellularized tissue. The collagen and elastin are determined via staining and quantified as they are majorly present ECM proteins in the esophagus and are structural proteins that help with the mechanical properties of the tissues. The retention of these ECM proteins proves the efficacy of the decellularization with the absence of nuclear material.

DNA quantification showed significant removal of resident cells of esophagus in decellularized tissue compared to native esophagus. About 97% reduction in DNA content proves the efficacy of the technique and the absence of DNA band in Figure 5 substantiates the result.

The SDS-PAGE analysis of the protein profiles of native and decellularized rat esophagus demonstrates the effectiveness of the decellularization process, depicted in Figure 6. The native esophagus (lane 2) showcases the diverse range of protein bands that includes both cellular and extracellular matrix components, whereas decellularized esophagus (lane 3) showed a reduction in the band intensity, indicating removal of cellular proteins and retention of EC proteins such as collagen and glycoproteins. The presence of these ECM components in the decellularized tissue provides biochemical stimulation for cell interaction and proliferation. Higher the content of marked ECM proteins, the interaction between the scaffold and cells will be better and provides a platform for enhanced gene expression and contribute to *in vivo* like microenvironment. Collagen bands range from 250 kDa to 150 kDa whereas sGAGs are seen around 75 kDa to 110 kDa.

sGAGs are family of linear and negatively charged polysaccharides that surround most cell types. In esophagus, the inner lining of mucosal squamous epithelium is glycoprotein rich and it also expresses a variety of keratin intermediate filament proteins that helps in providing structural integrity between the cells. The amount of sGAG in native and decellularized tissue was significantly similar, suggesting their retention post treatment. Thus, the chemical method of decellularization did not affect the sGAG content on the decellularized tissue. Though, there is an elastin decrease in dECM as compared to native tissue, the presence of these proteins will also contribute to cell proliferation and adherence.

AT-MSCs were selected for the cytotoxicity assay due to their ability to differentiate into epithelial cells [26], which are the resident cells of the esophagus. They are being used in esophageal tissue engineering due to their ability to differentiate into various cell types, including epithelial and mesenchymal lineages, and they can provide a source of cells capable of promoting tissue regeneration and integration [27]. AT-MSCs showcase immunomodulatory properties and secretion of growth factors that enhance therapeutic potentials and aid in repairing damaged tissues. AT-MSCs are metabolically active cells that make them excellent indicators of cytotoxic effects caused by material, drugs, or tissue degradation products. They showcase high sensitivity that allows accurate assessment of cell viability [28]. Hence, for cytotoxic study, it will be appropriate to use AT-MSCs on the decellularized esophagus. The AT-MSC derived from the adipose tissue exhibited the characteristics features of MSC and was screened according to the criteria of ISCT which states that MSCs must adhere to plastic surfaces under standard culture conditions. They should express specific surface markers, including CD73, CD90, and CD105 (≥95%), while lacking the expression of CD45, CD34, CD14, or CD11b, CD79a, or CD19, and HLA-DR (≤2%), unless induced by IFN-γ. Additionally, MSCs must demonstrate multipotent differentiation potential, meaning they should be capable of differentiating into osteogenic, adipogenic, and chondrogenic lineages. This is confirmed through specific staining techniques such as Alizarin Red S for osteogenesis, Oil Red O for adipogenesis, and Alcian Blue or Safranin O for chondrogenesis. These criteria ensure that the cells identified as MSCs possess the required phenotypic and functional characteristics [17]. AT-MSC showed trilineage differentiation (as shown in Figure 8(A)) and to adipocytes, osteocytes, and chondrocytes in comparison with control. The expression of 5% of CD 34 and CD 45 and 95% by CD 105 and CD 73 (Figure 8(B)) further strength the validation and as the cells possess mesenchymal stem cell properties [29]. Decellularization is an effective technique to obtain dECM proteins for scaffold fabrication. The scaffolds fabricated can be used for regeneration of the esophagus and in the defect area. In order to check if the decellularized tissue is biocompatible, cytotoxicity assay of the tissue is performed with AT-MSC. The conditioned media treated with decellularized tissue showed 89% cell viability proving the biocompatibility of decellularized tissue (Figure 9) [30].

SDS-PAGE was performed with different concentrations of solubilized decellularized tissue, confirming the retention of key ECM proteins such as collagen, laminin, fibronectin, and glycoprotein. Collagen bands range from 250 kDa to 150 kDa whereas sGAGs are seen around 75 kDa to 110 kDa. These proteins play essential roles in cell adhesion, proliferation, and tissue remodeling, making their preservation critical for scaffold functionality. The presence of major bands across different concentrations of solubilized dECM indicates effective retention of these ECM components post-solubilization. The band intensity decreased as the dECM concentration was reduced from 2% to 1%, reflecting lower protein content at lower concentrations (Figure 10). The successful solubilization of decellularized tissue demonstrates its potential for use as a hydrogel or in combination with other biomaterials for scaffold fabrication. The retention of ECM proteins enhances the bioactivity of these materials, supporting cell attachment, migration, and differentiation, which are crucial for esophageal tissue engineering.

The study underscores the significance of optimizing the decellularization technique to effectively remove the cellular components and preserve the essential ECM components. Our method ensures retention of ECM proteins, structural integrity, and biocompatibility. The solubilized dECM demonstrates versatility as a bioink, offering immense potential for fabricating 3D bioprinted scaffolds for esophageal regeneration.

## 6. Conclusion

Decellularization technique differs from species, organ and tissue to suit the application, the selection of cocktail of decellularizing agent is critical to obtain dECM. The ideal conditions for decellularization also retain the prominent proteins and to remove the genetic material to ensure the dECM is non-immunogenic and biocompatible. These resident ECM proteins contribute to regenerative and proliferative capability by providing biochemical cues to the cells. Hence, it is crucial to retain these proteins and maintain the function. Here, we have obtained dECM by using detergent-based method which utilizes CHAPS and SDS to completely remove the cellular content, that satisfies the condition of being non-immunogenic and that can be biocompatible, the option of application of the dECM as scaffold material is innumerable and can be expanded to their use as bioinks for 3D bioprinting or any other typer of scaffold fabrication.

## Supplementary Material

A02_UNSTAINED.fcs

A03_CD34.fcs

A07_CD73.fcs

A04_CD45.fcs

A06_CD105.fcs

## Data Availability

The authors confirm that the data supporting the findings of this study are available within the article

## Data availability statement

Data available within the article and the authors confirm that the data supporting the findings of this study are available within the article.
